# Coffee Consumption Modulates Amoxicillin-Induced Dysbiosis in the Murine Gut Microbiome

**DOI:** 10.3389/fmicb.2021.637282

**Published:** 2021-06-30

**Authors:** Emma Diamond, Katharine Hewlett, Swathi Penumutchu, Alexei Belenky, Peter Belenky

**Affiliations:** ^1^Department of Molecular Microbiology and Immunology, Brown University, Providence, RI, United States; ^2^TarMeta Biosciences Inc., Natick, MA, United States

**Keywords:** microbiome, gut, coffee, caffeine, antibiotics

## Abstract

The microbiome is essential for host health, and perturbations resulting from antibiotic use can lead to dysbiosis and disease. Diet can be a powerful modulator of microbiome composition and function, with the potential to mitigate the negative effects of antibiotic use. Thus, it is necessary to study the impacts of diet and drug interactions on the gut microbiome. Coffee is a commonly consumed beverage containing many compounds that have the potential to affect the microbiome, including caffeine, polyphenols, and fiber. We supplemented mice with caffeinated and decaffeinated coffee in conjunction with amoxicillin, and used 16S rRNA amplicon sequencing of fecal samples to investigate changes in diversity and composition of the murine fecal microbiome. We found that antibiotics, regardless of coffee supplementation, caused significant disruption to the murine fecal microbiome, enriching for Proteobacteria, Verrucomicrobia, and Bacteroidetes, but reducing Firmicutes. While we found that coffee alone did not have a significant impact on the composition of the fecal microbiome, coffee supplementation did significantly affect relative abundance metrics in mice treated with amoxicillin. After caffeinated coffee supplementation, mice treated with amoxicillin showed a smaller increase in Proteobacteria, specifically of the family Burkholderiaceae. Correspondingly we found that *in vitro*, *Burkholderia cepacia* was highly resistant to amoxicillin, and that it was inhibited by concentrations of caffeine and caffeinated coffee comparable to levels of caffeine in murine ceca. Overall, this work shows that coffee, and possibly the caffeine component, can impact both the microbiome and microbiome members during antibiotic exposure.

## Introduction

The human gut is home to trillions of bacteria which are vital for many host processes, including energy extraction from food, synthesis of important molecules, and protection from pathogens (Becattini et al., 2016). These microorganisms and their genetic content are collectively referred to as the gut microbiome. We now understand that the gut microbiota plays a key role in host health (Knight et al., 2017). Thus, studying the dynamic factors that affect microbiome composition and function is key to understanding and promoting health.

Most of the bacteria comprising the gut microbiome live in the colon, where they ferment substances indigestible to the host, such as fiber and polyphenols (Korpela, 2018). Fermentation leads to the production of short chain fatty acids (SCFAs) and this in turn increases the anaerobicity of the colon (Jha et al., 2019). Treatment with antibiotics decreases microbiome diversity and can cause antibiotic induced dysbiosis (AID). Under dysbiosis, the oxygen content of the gut increases, leading to decreased fermentation by obligate anaerobes and diminished SCFA production. This in turn decreases gut barrier integrity, which leads to inflammation and promotes the growth of facultative aerobic bacteria such as Proteobacteria (Jha et al., 2019). Some members of this phylum are opportunistic pathogens and are highly immunogenic, such as *Escherichia coli* and *Klebsiella* spp., and have the potential for expansion under dysbiotic conditions (Becattini et al., 2016; Kim et al., 2017; Zarrinpar et al., 2018).

Antibiotic use and AID not only lead to increased risk of secondary infections, but also are associated with chronic conditions including inflammatory bowel disease, asthma, diabetes, obesity, and other metabolic syndromes (Blaser, 2011; Cabral D. et al., 2020). As antibiotic use continues to rise worldwide, with tens of billions of daily doses taken each year, it is now more important than ever to investigate ways to prevent AID (Klein et al., 2018).

Several remedies for AID have been proposed and are in use, including the administration of probiotics and prebiotics. Prebiotics are substrates, such as fiber and polyphenols, that are selectively used by native bacteria to promote health, as opposed to probiotics which repopulate the gut with exogenous microbes (Preidis and Versalovic, 2009; Gibson et al., 2017; Rezende et al., 2021). Probiotics have the potential to increase abundance of the probiotic species rather than the original native population of the gut. In fact, supplementing the microbiome with probiotics after antibiotic use delays microbiome reconstitution as compared to spontaneous reconstitution (Suez et al., 2018). Thus, prebiotics may be a safer way to address AID.

Many substances have been studied for their potential as prebiotic compounds. Coffee is a common caffeinated beverage that contains dietary fibers like galactomannan and type II arabinogalactan, as well as polyphenols such as chlorogenic acid, which may act as an antioxidant (Gniechwitz et al., 2007; Liang and Kitts, 2015; Win et al., 2019). One study in rats showed that coffee stimulated smooth muscle contractility in the intestine in a dose-dependent manner (Hegde et al., 2019). Related studies have shown that this effect of caffeine can lead to a decreased transit time of nutrients in the gut, which has been shown to change microbiome composition by affecting water and nutrient availability throughout the gut (Brown et al., 1990; Kashyap et al., 2013). Caffeine has also been previously associated with a richer gut microbiome and may reduce the prevalence of inflammatory bacteria (Gurwara et al., 2019).

Several studies have explored the effects of coffee and its components on the gut microbiome. One study gave mice filtered coffee for 3 days and found that relative abundance of *Eubacteria, E. coli, Enterococcus*, and *Clostridium* decreased, while *Bifidobacteria* and *Lactobacillus* increased (Nakayama and Oishi, 2013). A similar study in humans found that after 3 weeks of consistent coffee intake, relative abundance of most bacterial species remained unchanged, with some increase in *Bifidobacteria* (Jaquet et al., 2009). While both coffee consumption and antibiotic administration have the potential to change the composition of the microbiome, the joint impacts of these perturbations are not established *in vivo*. Previous *in vitro* experiments have studied interactions between caffeine and antibiotics, finding that caffeine can lower the efficacy of many first-line antibiotics. Some possible explanations for this are competitive binding between caffeine and the antibiotic or formation of caffeine-antibiotic complexes (Olajuyigbe et al., 2017).

Here we to investigate the effects of coffee, both caffeinated and decaffeinated, on AID in the murine microbiome. We gave mice caffeinated and decaffeinated coffee for 12 days, in conjunction with amoxicillin for 7 days and analyzed gut microbiome samples by 16S rRNA sequencing. We found that compared to controls, mice treated with amoxicillin and given caffeinated coffee exhibited a decrease in the abundance of potentially pro-inflammatory bacteria, such as Proteobacteria.

## Materials and Methods

### Mouse Experiments

**FIGURE 1:**
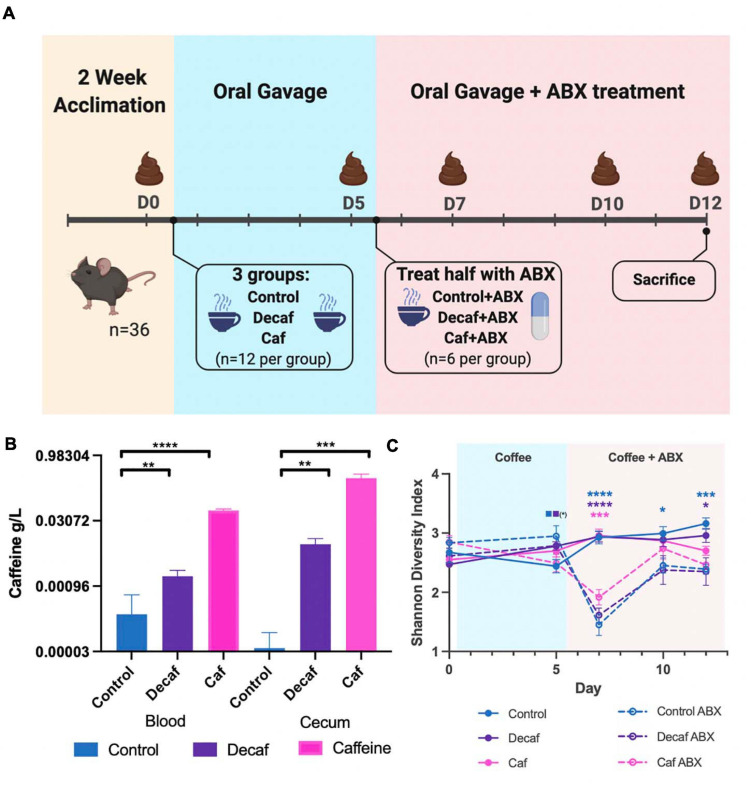
**(A)** Mouse experimental design and timeline. Figure was created with biorender.com. **(B)** Quantification of caffeine content in blood and cecum 1-h post-oral gavage (*n* = 6). **(C)** Alpha diversity of experimental groups as measured by the Shannon Diversity Index. Data are represented as mean ± standard deviation (SD). Significance between each group and its antibiotic counterpart is denoted by stars (*) with the color coordinating to control (ctrl, blue), decaffeinated coffee (decaf, purple), or caffeinated coffee (caf, pink). Significance between different experimental groups is denoted by squares color-coded according to the experimental groups being compared. Significance was determined by the Benjamini, Krieger and Yekutieli test to correct for False discoveries with adjusted *p*-value < 0.05. (*, 0.01 < *P* < 0.05; **, 0.001 < *P* < 0.01; ***, 0.0001 < *P* < 0.001; ****, *P* < 0.0001). Solid squares represent groups without antibiotics, open squares represent groups given antibiotics. Each comparison of two experimental groups has its corresponding significance denoted in stars (*) next to it. *n* = 6. For example, on Day 5, the solid blue square next to the solid purple square represents a significant difference between the control non-antibiotic group and the decaf non-antibiotic group, at a significance level of 1 star (shown in parentheses). On Day 12, the 3 blue stars represent a significant difference between the control non-antibiotic group and the control antibiotic group, at a significance level of 3 stars. The 1 purple star represents a significant difference between the decaf non-antibiotic group and the decaf antibiotic group at a significance level of 1 star.

All animal work was approved by Brown University’s Institutional Animal Care and Use Committee (IACUC) under protocol number 1706000283. Four-week-old female C57BL/6 mice were purchased from Jackson Laboratories (Bar Harbor, ME, United States) and given a 2-week habituation period immediately following arrival at Brown University’s Animal Care Facility, during which they were given standard chow (Laboratory Rodent Diet 5001, St. Louis, MO, United States) and water *ad libitum*. After habituation and for the duration of the experiment, mice were given daily oral gavage of caffeinated coffee (Starbucks Via Instant Italian Roast, 1.5 mg caffeine/400 μL), decaffeinated coffee (Starbucks Via Instant Decaf Italian Roast, 0.14 mg caffeine/400 μL), or water (400 μL) (*n* = 12 per group), per the protocol described in Nakayama and Oishi (2013). After 5 days of gavage, half of each group (*n* = 6 per group) were administered antibiotics in the drinking water (amoxicillin 25 mg/kg/day) for 7 days. Oral gavage of coffee or water was continued throughout antibiotic treatment. Mice were sacrificed on day 12 of the experiment. Fecal samples were collected for 16S rRNA analysis on days 0 (before first oral gavage), 5 (before first antibiotic treatment), 7, 10, and 12 (Figure 1A).

### Mass Spectrometry for Caffeine Quantification

Eighteen additional mice were randomly assigned to receive oral gavage of caffeinated coffee, decaffeinated coffee, or water control (*n* = 6). One hour after gavage, mice were sacrificed and their blood and ceca were removed. 30 μL of plasma was extracted from the blood and combined with 94 μL of methanol and 6 μL of 0.01 mg/mL internal standard (IS) caffeine solution (caffeine labeled with two C13, MW 188). For every 1 mg of cecum, 10 μL of methanol with IS caffeine solution was added. Each mixture was vortexed for 5 min at 1,175 rpm, then centrifuged for 5 min at 17,900 × *g*. 15 μL of supernatant was removed for analysis.

Caffeine in biological samples after spiking with IS and extraction was quantitated with HPLC-MS on an Ultimate 3000, Dionex coupled to Q Executive Classic (Thermo) with ESI interface. Data acquisition and processing was performed by Excalibur software. The chromatographic separation was achieved on a Xselect CSH C18 2.5 μm; 2.1 × 30 mm (Waters, Milford, MA, United States), at 60°C. Mobile phase consisted of water for phase A and 50/50 methanol/acetonitril for phase B, both containing 0.2% FA. Separation was optimized using a fast gradient method with mobile phase A/B set to 90%/10% from 0.00 to 0.20 min and 10%/90% from 0.21 to 1.4 min and then back after 1.7 min for equilibration at 90%/10% from 1.7 to 6.00 min. with the flow 0.35 ml/min. The mass spectrometer was operated in the positive ion mode in Full MS; at resolution 70,000; AGT target 5 E5 and Scan range 170–200 m/z. Spray voltage and source temperature were set at 3,500 volts, and 320°C, respectively. CAF-2C13 was the IS used for quantification of CAF.

### Nucleic Acid Extraction and Quantification

Total nucleic acids (DNA) were extracted from samples using the ZymoBIOMICS DNA Miniprep Kit from Zymo Research (R2002, Irvine, CA, United States) using the extraction protocol as per the manufacturer instructions. Total DNA was eluted in nuclease-free water and quantified using the dsDNA-HS on a QubitTM 3.0 fluorometer (Thermo Fisher Scientific, Waltham, MA, United States) before use in amplicon preparations.

### 16S rRNA Amplicon Preparation and Sequencing

The 16S rRNA V4 hypervariable region was amplified from total DNA using the barcoded 515F forward primer and the 806R reverse primers from the Earth Microbiome Project (Thompson et al., 2017). Amplicons were generated using 5X Phusion High-Fidelity DNA Polymerase under the following cycling conditions: initial denaturation at 98°C for 30 s, followed by 25 cycles of 98°C for 10 s, 57°C for 30 s, and 72°C for 30 s, then a final extension at 72°C for 5 min. After amplification, samples were visualized via gel electrophoresis and pooled in equimolar amounts. The pooled amplicon library was submitted to the Rhode Island Genomics and Sequencing Center at the University of Rhode Island (Kingston, RI, United States) for sequencing on the Illumina MiSeq platform. Amplicons were paired-end sequenced (2 × 250 bp) using the 600-cycle kit with standard protocols. We obtained an average of 27,563 reads per sample. Raw reads were deposited in the NCBI Sequence Read Archive (SRA) under BioProject PRJNA682275.

### Analysis of 16S rRNA Sequencing Reads

Raw 16S rRNA reads were subjected to quality filtering, trimming, de-noising with DADA2 (Callahan et al., 2016) (via q2-dada2), and merging using the Qiime2 pipeline (version 2019.10) (Bolyen et al., 2019). Ribosomal sequence variants were aligned with mafft (Katoh et al., 2002) (via q2-alignment), and phylogenetic tree construction was done with fasttree2 (Price et al., 2010) (via q2-phylogeny). Taxonomic assignment was conducted using the pre-trained Naive Bayes classifier and the q2-feature-classifier (Bokulich et al., 2018a) trained on the SILVA 132 99% database (Quast et al., 2013). Alpha diversity (Shannon, Faith’s phylogenetic diversity) and beta diversity (Bray-Curtis dissimilarity) (Bray and Curtis, 1957; Bokulich et al., 2018b) were calculated using the phyloseq package (version 1.30.0) in R (version 3.6.2) (Faith, 1992; Lozupone and Knight, 2005; Lozupone et al., 2007). Significance was determined by the Benjamini, Krieger and Yekutieli test to correct for False discoveries with adjusted *p*-value < 0.05 (Benjamini and Hochberg, 1995). Linear Discriminant analysis was conducted using the LEfse package (version 1.0) on the galaxy server^1^ (Segata et al., 2011; Afgan et al., 2018). Raw 16S rRNA reads were deposited in the NCBI Sequence Read Archive (SRA) under BioProject number PRJNA682275.

### Minimal Inhibitory Concentration Determination

Minimal inhibitory concentrations (MICs) were determined using the broth dilution method (Wiegand et al., 2008). Overnight cultures of *B. cepacia* or *E. coli* were diluted 100-fold into TSB and LB media, respectively. Cultures were incubated with various concentrations of caffeine (Sigma-Aldrich, St. Louis, MO, United States), caffeinated coffee or decaffeinated coffee (Starbucks). Amoxicillin (Sigma-Aldrich, St. Louis, MO, United States) was added at varying concentrations to cell culture media and serially diluted two-fold. Cells were then incubated aerobically at 37°C until controls reached their maximum OD_600_ reading (18 h).

### *B. cepacia* Growth Rate Determination With Caffeinated and Decaffeinated Coffee

Overnight cultures of *B. cepacia* grown in TSB were pelleted and resuspended in 1X PBS. Cells were subsequently diluted to an optical density at 600 nm (OD_600_) of approximately 0.01 into TSB supplemented with caffeinated coffee, decaffeinated coffee or caffeine. Cells were incubated at 37°C under aerobic conditions and growth was monitored by taking OD_600_ readings at 10-min intervals. To determine the doubling time in each condition, the growth curves were fitted to an exponential growth function using default settings in Prism (version 8.4.2).

## Results

### Oral Gavage With Coffee Leads to Significant Increases in Murine Blood and Cecum Caffeine Levels

To determine the effect of coffee and its components on the murine gut microbiome, female C57BL/6J mice were randomly assigned to receive oral gavage of caffeinated coffee (caf), decaffeinated coffee (decaf), or water (control) (*n* = 12) (Figure 1A). Mice given caffeinated coffee received 1.5 mg caffeine at each gavage (per manufacturer-reported caffeine content), equivalent to 3 cups of coffee according to FDA human to animal dose conversion guidelines (Nair and Jacob, 2016). Mice given an equal volume of decaffeinated coffee received 0.14 mg caffeine at each gavage. After 5 days, half of the mice from each group additionally received amoxicillin in their drinking water (*n* = 6) for 7 days (Cabral D. et al., 2020). Fecal samples were taken on days 0, 5, 7, 10, and 12 for 16S rRNA amplicon sequencing of the fecal microbiome. We utilized mass spectrometry to determine the maximum level of caffeine present in the ceca and blood of mice 1-h post-oral gavage, using 6 additional mice (Teekachunhatean et al., 2013). Caffeine supplementation led to a significant increase in cecum and blood caffeine levels (Figure 1B). Blood caffeine levels were approximately 0.0530 g/L and cecum levels were approximately 0.293 g/L. While these likely represent peak caffeine levels, based on the rapid metabolic activity of mice we expect that this level would drop significantly between gavages (Teekachunhatean et al., 2013). This also indicates that while some caffeine was absorbed into the bloodstream, the majority of the caffeine was unabsorbed and available to the microbiota. As described above, coffee is a complex mixture of fibers and polyphenols and, like caffeine, these molecules would also become available to gut microbes.

### Antibiotics and Coffee Supplementation Lead to Changes in Bacterial Diversity in the Fecal Microbiome

The 16S rRNA v4 hypervariable region was sequenced to determine the bacteria present in the fecal microbiome of mice. Overall, the average sequencing depth was approximately 27,563 and the data were analyzed using Qiime2 to determine metrics of bacterial diversity as well as composition. We did not detect an impact of coffee administration on the diversity of the fecal microbiome as measured by the Shannon Diversity Index over the entire length of the experiment (Figure 1C). Conversely, antibiotics caused a significant drop in microbial diversity, as shown by the significant decrease in diversity in the antibiotic-supplemented groups at 2 days post antibiotic administration (Figure 1C). The caf group showed a smaller antibiotic-induced decrease in diversity (*p* = 0.064), potentially indicating that caffeinated coffee supplementation in this amount may reduce the drop in microbial diversity.

**FIGURE 2:**
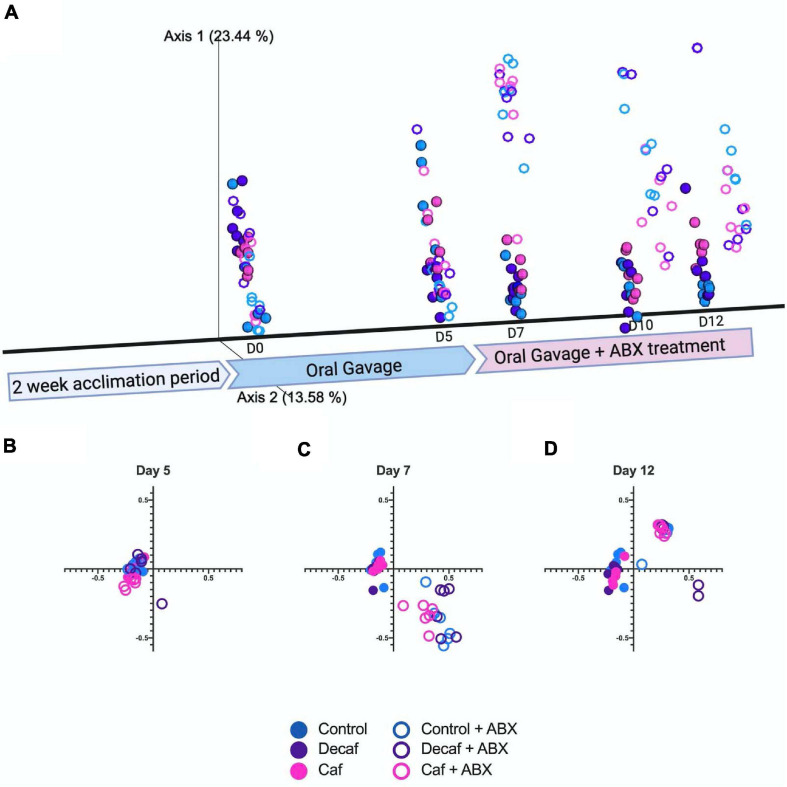
**(A)** Beta diversity of experimental groups over time represented on a three-dimensional principal coordinate analysis plot. Figure was created with biorender.com. **(B)** Beta diversity metrics using Bray Curtis Distance Matrices at 5 days of coffee gavage, **(C)** 2 days of antibiotic treatment and 7 days coffee gavage, **(D)** 7 days of antibiotic treatment and 12 days coffee gavage; *n* = 6.

We used the Bray-Curtis beta-diversity metric to assess the degree of dissimilarity between our samples. After 5 days of oral coffee gavage, caf and decaf mice did not cluster separately from mice gavaged with the vehicle. This indicates that compounds in coffee or caffeine alone do not significantly alter the overall microbiome composition (Figures 2A,B). As other studies have found, antibiotics led to significant changes in microbiome composition in mice, regardless of supplementation (Cabral D. et al., 2020; Cabral D. J. et al., 2019). Over the course of the experiment, our samples formed clusters mainly based on amoxicillin treatment (Figures 2C,D).

### Antibiotics Alter Fecal Microbiome Composition by Reducing Firmicutes and Increasing Bacteroidetes, Proteobacteria, and Verrucomicrobia

**FIGURE 3:**
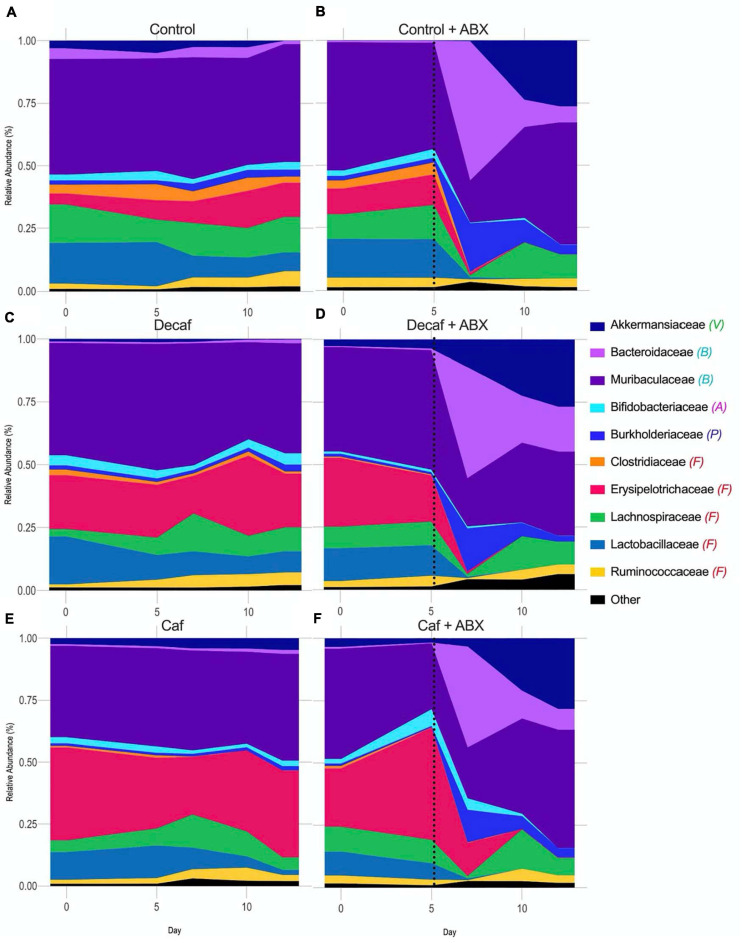
Relative abundance of bacterial families over the course of the experiment. Phylum denoted by: V, Verrucomicrobia; B, Bacteroidetes; A, Actinobacteria; P, Proteobacteria; F, Firmicutes. Dotted line denotes beginning of antibiotic treatment. **(A)** Control, **(B)** Control + amoxicillin days 5–12, **(C)** Decaf, **(D)** Decaf + amoxicillin days 5–12, **(E)** Caf, **(F)** Caf + amoxicillin days 5–12; *n* = 6.

While the overall beta-diversity indicated that antibiotic exposure had a profound impact on microbial composition, it appears that coffee had a very modest impact on composition in the broad sense. To directly determine which microbial families were affected throughout the experiment, we plotted their relative abundance over time and utilized LEfSe to determine which bacteria were significantly associated with each experimental group. From visual analysis of the data, we found that the greatest perturbation in composition occurred 2 days after antibiotic exposure (day 7) (Figure 3 and Supplementary Tables 1, 2). After this point, the microbiome began to return to a new altered baseline as antibiotic therapy progressed. For example, *Burkholderiaceae* from the Proteobacteria phylum had a peak of expansion on day 7, and then returned back to baseline. Similarly, *Bacteroidaceae* from the Bacteroidetes phylum peaked at day 7. This trend was inverted for *Muribaculaceae*, which collapsed at day 7. Finally, *Akkermansiaceae* began to increase at day 7 and then expanded throughout the rest of the experiment. Looking at these data, we can also see that supplementing with caffeinated or decaffeinated coffee has an impact on the intensity of these changes. For example, the expansion of *Burkholderiaceae* appears to be greater in the control and decaf groups than in the caf group (Figure 3 and Supplementary Table 1).

**FIGURE 4:**
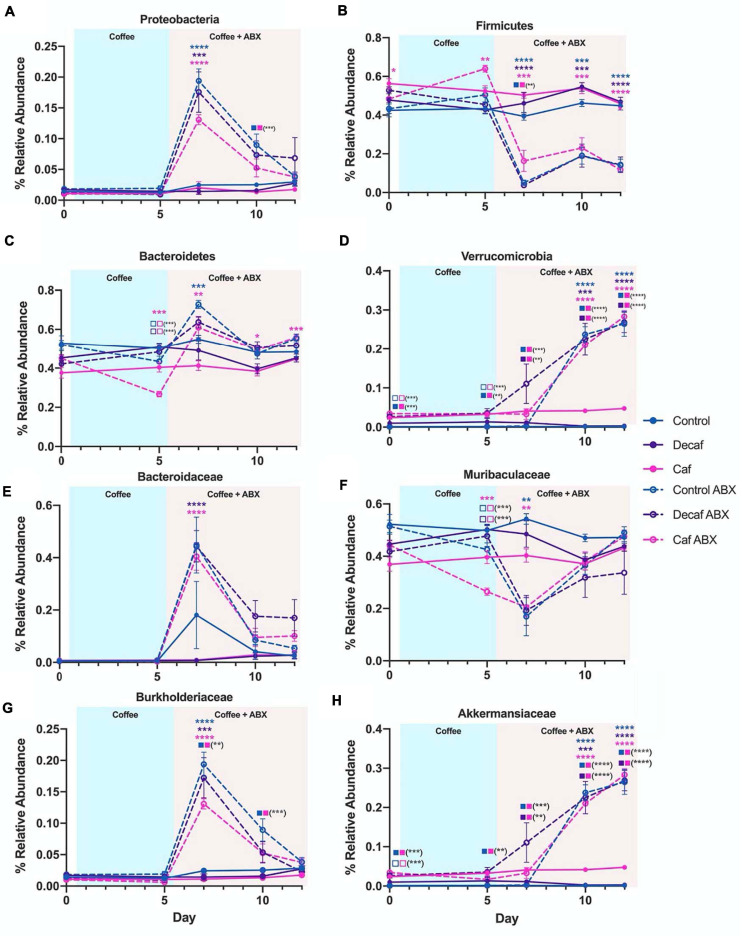
**(A–H)** Relative abundance of notable phyla and families over the course of the experiment. Data are represented as mean ± SD. Significance was determined by the Benjamini, Krieger and Yekutieli test to correct for False discoveries with adjusted *p*-value < 0.05 (*, 0.01 < *P* < 0.05; **, 0.001 < *P* < 0.01; ***, 0.0001 < *P* < 0.001; ****, *P* < 0.0001). Significance between each experimental group and its antibiotic counterpart is denoted by stars (*) with the color coordinating to control (ctrl, blue), decaffeinated coffee (decaf, purple), or caffeinated coffee (caf, pink). Significance between different experimental groups is denoted by squares color-coded according to the experimental groups being compared. Solid squares represent groups without antibiotics, open squares represent groups given antibiotics. Each comparison of two experimental groups has its corresponding significance denoted in stars (*) next to it. For example, in the Bacteroidetes graph **(C)** on Day 5, the 3 pink stars represent a significant difference between the caf no-antibiotic group and the caf antibiotic group, at a significance level of 3 stars. Below that, the open blue square next to the open pink square represents a significant difference between the control antibiotic group and the caf antibiotic group, at a significance level of 3 stars (shown in parentheses). Below that, the open purple square next to the open pink square represents a significant difference between the decaf antibiotic group and the caf antibiotic group, at a significance level of 3 stars (shown in parentheses).

In order to gain an appreciation for the statistical significance of these changes, we utilized LEfSe to determine drug-induced and supplement-induced changes at the phylum and family levels on day 7, since this was the time point with the greatest perturbations. In all groups, amoxicillin exposure caused an increase in Proteobacteria (*t*-test *P* < 0.05; LEfSe Kruskal–Wallis alpha = 0.05, LDA Threshold = 2.0) (Figure 4A and Supplementary Figures 3A,C,E). This expansion was matched by a reduction in Firmicutes (Figure 4B). While Proteobacteria expanded in all treatment groups, the level of expansion differed based on supplementation. Comparing the control antibiotic (control ABX) treated group to the caffeinated coffee antibiotic (caf ABX) treated group, we found that Proteobacteria were significantly associated with the control ABX group (LEfSe) (Figure 4A and Supplementary Figure 3J). This differential association was not observed when we compared the decaf antibiotic (decaf ABX) group to the control ABX group (LEfSe) (Supplementary Figure 3H), possibly indicating that caffeinated but not decaffeinated coffee reduced the antibiotic-induced expansion in Proteobacteria. Bacteroidetes relative abundance was significantly higher in the control ABX and caf ABX mice 2 days after amoxicillin treatment as compared to their non-antibiotic counterparts. The caf ABX group was the only one to stay significantly elevated as compared to its control counterpart throughout the rest of the experiment (*t*-test *P* < 0.05) (Figure 4C). Visually, Verrucomicrobia began to expand at day 7, although this expansion appeared to be delayed in the control group. This perceived delay is followed by a significant association of Verrucomicrobia with the decaf ABX group, but not the control ABX group at day 7 (LEfSe) (Figures 4D,H and Supplementary Figure 3G).

Biomarker analysis using LEfSe at lower taxonomic levels showed that the strongest associations observed at the phylum level could be traced down to specific families (Supplementary Figure 3). Within the Bacteroidetes phylum, we found that an increase in the relative abundance of *Bacteroidaceae* was significantly associated with all three antibiotic-treated groups compared to their non-antibiotic counterparts at day 7 (LEfSe) (Figure 4E and Supplementary Figures 3B,D,F). This expansion was reversed for *Muribaculaceae*, which was significantly associated with the non-antibiotic treated groups at the same time point (LEfSe) (Figure 4F and Supplementary Figures 3B,D,F). The only family found to be significantly different between experimental groups for Proteobacteria was *Burkholderiaceae*, which was significantly increased in the antibiotic-treated groups as compared to their non-antibiotic counterparts at day 7 (*t*-test *P* < 0.05; LEfSe) (Figure 4G and Supplementary Figures 3B,D,F). This likely explains the differences in Proteobacteria observed at the phylum level. As we observed in Proteobacteria, this antibiotic-related increase in *Burkholderiaceae* was not significantly associated with the caf group, but was significant in the control and decaf group (LEfSe) (Figure 4G and Supplementary Figure 3I). As expected, the expansion in *Burkholderiaceae* was not impacted by decaffeinated coffee (Figure 4G and Supplementary Figure 3H). Within the Verrucomicrobia phylum, *Akkermansiaceae* was significantly associated with the caf ABX and decaf ABX groups as compared to the control ABX group at Day 7 (LEfSe) (Figure 4D and Supplementary Figures 3B,D,F). By day 10, all three antibiotic-treated groups had significantly more *Akkermansiaceae* than their non-antibiotic counterparts, confirming the delayed expansion in the control group observed at the phylum level (*t*-test *P* < 0.05; LEfSe) (Figure 4H and Supplementary Figures 4A–C).

### *Burkholderia cepacia* Is Resistant to Amoxicillin

**FIGURE 5:**
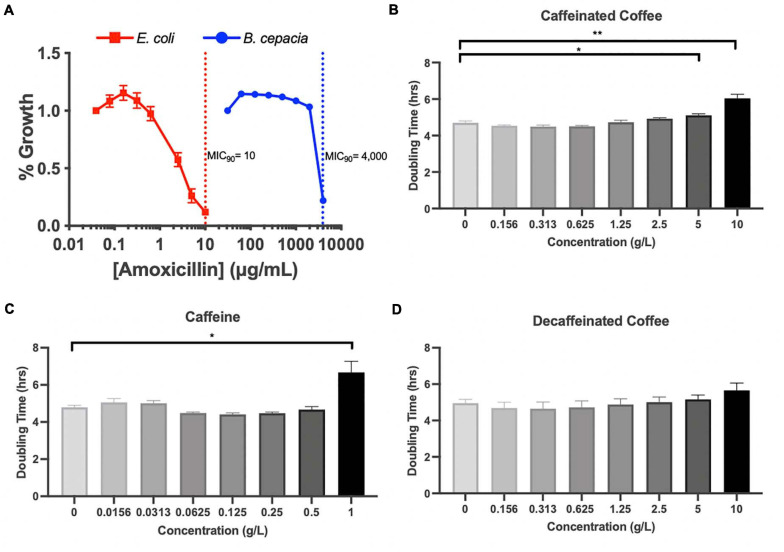
**(A)** MIC assessment of *B. cepacia*, *E. coli* with amoxicillin in TSB or MGAM, respectively, containing varying concentrations of amoxicillin. MIC_90_ indicated by dotted lines. Doubling time calculated from growth curves of *B. cepacia* cultured in TSB with varying concentrations of **(B)** caffeinated coffee, **(C)** caffeine, and **(D)** decaffeinated coffee; *n* = 3 (*, 0.01 < *P* < 0.05; **, 0.001 < *P* < 0.01; unpaired *t* test).

Because Proteobacteria are strongly associated with dysbiotic microbiomes (Becattini et al., 2016), we further investigated the impact of coffee and caffeine on *Burkholderiaceae in vitro.* 16S analysis does not allow us to accurately characterize bacteria down to the species level, so we chose to utilize *B. cepacia* as a model from the *Burkholderiaceae* family to investigate the impact of coffee and amoxicillin. *B. cepacia* is a common member of the murine gut microbiome and has been associated with inflammation (Armstrong et al., 2019). It is also an opportunistic pathogen found in the lungs of cystic fibrosis patients (Scoffone et al., 2017). We determined the minimal inhibitory concentration (MIC_90_) of amoxicillin for *B. cepacia*, and found it to be 4000 μ/mL, indicating that it is essentially resistant to this drug. As a control, we determined the MIC_90_ of *E. coli* was much lower, at 10 μ/mL (Figure 5A). Other studies have also found that *B. cepacia* is highly resistant to amoxicillin treatment (Everaert and Coenye, 2016). This resistance is a possible explanation for the significant increase in *Burkholderiaceae* relative abundance immediately after amoxicillin treatment in all three groups (Figure 4G). However, this does not explain the fact that *Burkholderiaceae* did not expand to the same extent in the caf ABX group as compared to the decaf and control ABX groups. Other data indicate that some species of *Burkholderiaceae* have the capacity to degrade caffeine, so we next investigated the impact of caffeine on *B. cepacia’s* growth rate (Win et al., 2019).

We measured the doubling time of *B. cepacia in vitro* when exposed to increasing concentrations of caffeinated coffee, decaffeinated coffee, and pure caffeine. We found that the doubling time of *B. cepacia* was significantly greater when exposed to the two highest concentrations of caffeinated coffee, as compared to the control (6.7 h vs. 4.8 h) (Figure 5B). This significant inhibition in growth also occurred at the highest concentration of pure caffeine (Figure 5C). We did not observe any significant changes in doubling time at any concentration of decaffeinated coffee, although there was an overall upward trend (Figure 5D). Although some species of *Burkholderiaceae* may utilize caffeine, because these experiments were performed in rich media, the pure caffeine is likely not acting as a primary carbon source. In fact, other work shows that high levels of caffeine may be inhibitory to some species of *Burkholderiaceae*, as reflected in our findings (Win et al., 2019). Thus, *in vitro*, we see that caffeine and caffeinated coffee are factors that can affect bacterial behavior. Overall, these data indicate that coffee, and even possibly caffeine, may have an inhibitory impact on these bacteria in the gut, possibly explaining the observed reduction in relative abundance during the antibiotic-induced bloom.

## Discussion

Coffee is made up of several components that individually have the potential to change the gut microbiome, including caffeine, polyphenols, and fiber (Gniechwitz et al., 2007; Liang and Kitts, 2015). Previous studies have investigated the effect of coffee on the microbiome, but to our knowledge there has not been extensive research investigating the combined effects of coffee and antibiotics on the gut microbiome (Jaquet et al., 2009; Nakayama and Oishi, 2013; Gurwara et al., 2019). Food and drug interactions are important to study, as they have the potential to impact microbiome composition, function, and host health in ways that cannot necessarily be predicted (Cabral D. et al., 2020; Cabral D. J. et al., 2019). Coffee and antibiotics are both commonly consumed, presumably together, and therefore it is important to investigate their combined effects on the gut microbiota (Klein et al., 2018).

In this study, we found that neither caffeinated nor decaffeinated coffee alone has a major impact on the diversity or composition of the murine fecal microbiome. However, we did find that coffee supplementation has some impact on the taxonomic composition of the microbiome in mice treated with amoxicillin, although this impact is not reflected in statistically significant changes in alpha or beta diversity. We found that, according to LEfSe analysis, *Akkermansiaceae* was significantly associated with both coffee groups in mice treated with antibiotics, and not with the control mice. This may indicate that a component common to caffeinated and decaffeinated coffee is directly or indirectly affecting this population. In addition, we observed fewer Proteobacteria in amoxicillin-treated mice given caffeinated coffee in the relative abundance analysis, as compared to mice given antibiotics and decaffeinated coffee or the vehicle (Figures 3B,D,F), an observation which was confirmed by LEfSe analysis of significance. We tested LDA effect size at the phylum and family level and determined that there were significant changes at the level of the Proteobacteria phylum and the *Burkholderiaceae* family, which changed in response to amoxicillin and coffee treatment. Proteobacteria are associated with inflammation and dysbiosis due to antibiotics, so a reduction in this phylum may represent a beneficial effect of caffeinated coffee on the microbiome. Some species of *Burkholderiaceae* that reside in the gut have pathogenic potential, and *B. cepacia* has been associated with irritable bowel syndrome (Rizzatti et al., 2017; Armstrong et al., 2019). *B. cepacia* is also an important opportunistic pathogen found in cases of cystic fibrosis (Scoffone et al., 2017).

While coffee alone did not seem to impact overall microbial composition, some differences could be observed. However, the main difference in *Akkermansiaceae* levels appears to be an artifact of individual differences in baseline microbiota composition. *Akkermansiaceae* levels were significantly higher in mice given caffeinated coffee in comparison to mice given decaffeinated coffee or the vehicle in the absence of amoxicillin. However, at day 0 before any coffee supplementation, these mice had more of this bacterial family than controls, so this finding may not be attributable to coffee supplementation. While we did find some other significant differences in relative abundance of certain bacterial phyla and families between coffee experimental groups, these often only were significant for a single time point and did not remain consistent throughout the rest of the experiment.

Using MIC assays and growth curves, we further investigated the response of *B. cepacia* to amoxicillin and coffee. As we expected based on previous studies, *B. cepacia* was highly resistant to amoxicillin (Everaert and Coenye, 2016). We also found that it was significantly inhibited by caffeinated coffee and pure caffeine at concentrations slightly above the maximum concentration detected in the ceca of our mice. While this result does not directly demonstrate that *Burkholderiaceae* inhibition *in vivo* is related to caffeine content, this conclusion is still possible since the concentration dependence and dynamics of inhibition may be different in the natural environment of the cecum compared to artificial media. In addition, the *in vitro* doubling time was measured over a relatively short period of time and a single exposure to the caffeine, whereas in the mouse, the microbiome was exposed to caffeine multiple times and over a much longer period of time. The metabolic environment is a known determinant of antimicrobial susceptibility to antibiotics and other toxins (Allison et al., 2011; Belenky et al., 2015; Lobritz et al., 2015; Cabral D. et al., 2020). This indicates caffeine has the potential to slow the growth of *B. cepacia in vitro*, which may explain why there were fewer *Burkholderiaceae* in the caffeinated coffee-supplemented mice after antibiotic treatment. However, caffeinated and decaffeinated coffee do not differ exclusively in caffeine content. Our coffee was decaffeinated by washing coffee beans with methylene chloride and then roasting the coffee to evaporate all methylene chloride out of the product, a common decaffeination method (How Is Coffee Decaffeinated? | Britannica, 2020). Caffeine is soluble in methylene chloride, along with chlorogenic acid, one of the main polyphenols in coffee, meaning decaffeinated coffee has fewer polyphenols. Chlorogenic acid has the potential to act as an antioxidant and can be metabolized by gut bacteria (Couteau et al., 2001; Liang and Kitts, 2015). Higher levels of chlorogenic acid in caffeinated coffee could also partly explain the different levels of Proteobacteria seen in our mice after antibiotic exposure. Thus, the differences observed between caffeinated and decaffeinated coffee may be the result of other components of coffee besides caffeine. In addition, physiological effects of coffee consumption such as decreased transit time of nutrients in the gut could be occurring *in vivo* leading to changes in microbial abundance (Brown et al., 1990; Kashyap et al., 2013; Gurwara et al., 2019).

The work presented here is an early step in determining the interaction between coffee consumption and antibiotic-induced dysbiosis. Additionally, the study presented here is the first, to our knowledge, to show the combined effects of antibiotics and coffee consumption on the gut microbiome *in vivo.* Based on these results, we may make some predictions to drive future research. Our results lead us to conclude that consuming even a high amount of coffee does not significantly impact the murine microbiome, and if this translates to humans, indicate that coffee is not a source of microbiome disruption. Our data also show that while amoxicillin clearly disturbs the microbiome, adding coffee consumption does not drastically exacerbate this perturbation. In fact, coffee supplementation might even be beneficial to the gut, as shown by the reduction in the bloom of Proteobacteria in the caffeinated coffee-treated groups. Since coffee is one of the most polyphenol-rich and caffeine-rich foods we consume (Leonard et al., 2020), these results may also give us information about the extent to which polyphenols, particularly chlorogenic acid, and caffeine individually impact the composition of the microbiome. This is an important step in increasing our knowledge of dietary means to prevent or treat antibiotic-induced dysbiosis and its associated diseases, an increasing problem as antibiotic use continues to rise worldwide (Klein et al., 2018).

Overall, we found that coffee alone has very little impact on the diversity and structure of the mouse fecal microbiome. This finding is surprising for several reasons. Firstly, we administered a relatively large amount of coffee for 12 days, and other studies have found that coffee and its components can significantly alter the composition of the microbiome (Jaquet et al., 2009; Nakayama and Oishi, 2013). Additionally, coffee can directly impact gastrointestinal physiology (González et al., 2020). For example, in a rat model, coffee was shown to stimulate intestinal smooth muscle contractility in a dose-dependent manner (Hegde et al., 2019). The reduction of colonic transit time has the capacity to shape the microbiota, as the speed at which substances travel through the gut can change water and nutrient availability, as well as the rate of luminal washout (Müller et al., 2018). *In vitro* models have also confirmed that decreasing transit time can change microbial composition, leading to a decrease in certain bacterial populations (Child et al., 2006). Fiber, a component of coffee, also has laxation effects in the gut (Müller et al., 2018). However, we did not observe a change in microbiome composition despite these potential effects. Broadly speaking, this study may indicate that one of the most widely consumed substances in the world does not significantly impact microbiome composition (Manchón et al., 2013).

There are some limitations to this study that must be taken into account when interpreting the results and designing future experiments. While we did not observe major changes in microbial composition or diversity in mice supplemented with coffee alone, we did observe some significant differences in mice supplemented with coffee and treated with antibiotics for a few phyla. We found that the microbiome was the most affected on the second day of antibiotic treatment (day 7), and a general but incomplete recovery in diversity and composition was observed through the end of the experiment (day 12). Conducting statistical testing on Shannon diversity using the Holm-Šídák method revealed that decaf (adjusted *p*-value = 0.0202), control ABX (adjusted *p*-value = 0.0237), and caf ABX (adjusted *p*-value = 0.0307) were still significantly different at day 12 compared to day 0. In addition to these diversity differences, we also observed taxonomic differences between the treated and untreated groups at day 12 using LEfSe (Supplementary Figure 6). Due to this partial recovery and the robust changes observed early on, we focused on day 7 to analyze significant changes in microbial composition due to antibiotics. While the initial impact is clearly important for long-term disruption of the microbiome, this later time point should also be considered as it can impact recovery after treatment.

The effects of coffee on the microbiome may differ depending on the method of coffee production, the type of beans used, whether it is brewed or instant coffee, and the method of decaffeination, as all of these can affect the levels of caffeine, polyphenols, and fiber within coffee. We used only one brand and type of coffee, Starbucks instant coffee, and thus captured only the impact of this specific formulation. Future studies could investigate the impact of coffee’s individual components, especially polyphenols, on the fecal microbiome. In addition, humans typically consume coffee intermittently throughout the day, unlike through a single oral gavage dose that we utilized in our mice. This may affect how well our results can be extrapolated to humans. We used only one concentration of caffeine and amoxicillin in our *in vivo* experiments, so nuances in the effect of concentration on the microbiome may have been missed. There was also no recovery period for mice treated with antibiotics, so we were unable to determine if there was any difference in microbiome recovery for mice given different treatments. We were also limited in our studies due to the fact that 16S analysis cannot accurately tell us the genus or species of the bacteria we observed, so we could not get a clear picture of the microbiome at lower taxonomic levels than those shown here. Since the microbiome cannot solely inform us of the state of someone’s health, it would be interesting to see future studies on host physiology post-coffee and antibiotic administration. Such studies could include histology of the intestine to look for changes in gut permeability induced by treatment and qPCR to determine expression levels of important gut barrier genes.

In conclusion, as antibiotic use continues to be a necessary treatment, there remains a need to investigate dietary components with potential to impact antibiotic-induced dysbiosis, such as coffee and caffeine. The results of this study show that coffee consumption alone does not perturb the microbiome within the timeframe of the study. In addition, we found that in the context of antibiotic administration, coffee consumption does impact the response of the microbiome to amoxicillin. Although the observed changes at the taxonomic level were not dramatic, they may point to a potentially beneficial modulation. Thus, we believe that this work should be followed up with additional research looking at other drugs, timing of administration, and the *in vitro* impact of coffee and caffeine on other bacterial taxa.

## Data Availability Statement

The datasets presented in this study can be found in online repositories. The names of the repository/repositories and accession number(s) can be found below: https://www.ncbi.nlm.nih.gov/, PRJNA682275.

## Ethics Statement

The animal study was reviewed and approved by Brown University’s Institutional Animal Care and Use Committee (IACUC) under protocol number 1706000283.

## Author Contributions

PB, ED, KH, and SP conceptualized the project and assisted in experimentation, writing, and editing. AB conducted MS analysis. All authors have reviewed and approved the manuscript for final version.

## Conflict of Interest

AB was employed by the company TarMeta Biosciences Inc. This company had no financial stake or involvement in the project. The remaining authors declare that the research was conducted in the absence of any commercial or financial relationships that could be construed as a potential conflict of interest.

## Footnotes

1 https://huttenhower.sph.harvard.edu/galaxy/

## Supplementary Material

Supplementary Figure 1 Phylogenetic diversity of experimental groups as measured by the Faith’s Phylogenetic Diversity Index. Data are represented as mean ± standard deviation (SD). Significance was determined by the Benjamini, Krieger and Yekutieli test to correct for False discoveries with adjusted *p*-value < 0.05. (*, 0.01 < *P* < 0.05; **, 0.001 < *P* < 0.01; ***, 0.0001 < *P* < 0.001; ****, *P* < 0.0001). Significance between each group and its antibiotic counterpart is denoted by stars (*) with the color coordinating to control (ctrl, blue), decaffeinated coffee (decaf, purple), or caffeinated coffee (caf, pink). Significance between different experimental groups is denoted by squares color-coded according to the experimental groups being compared. Solid squares represent groups without antibiotics, open squares represent groups given antibiotics. Each comparison of two experimental groups has its corresponding significance denoted in stars (*) next to it. *n* = 6.

Supplementary Figure 2 Relative abundance of bacterial phyla over the course of the experiment. Dotted line denotes beginning of antibiotic treatment. **(A)** Control, **(B)** Control + amoxicillin days 5–12, **(C)** Decaf, **(D)** Decaf + amoxicillin days 5–12, **(E)** Caf, **(F)** Caf + amoxicillin days 5–12. *n* = 6.

Supplementary Figure 3 Linear discriminant analysis effect size (LEfSe) analysis between treatment groups on day 7 at the phylum level **(A,C,E,G,I)**, and at the family level **(B,D,F,H,J)**. Histogram of LDA scores plotted on a log 10 scale.

Supplementary Figure 4 **(A–C)** Linear discriminant analysis effect size (LEfSe) analysis between antibiotic treated and control groups on day 10 at the family level. Histogram of LDA scores plotted on a log 10 scale.

Supplementary Figure 5 *Burkholderiaceae* relative abundance, with control and antibiotic samples interleaved by ranked abundance of each taxon. Means represented by dotted (ABX) or dashed (Control) lines. Relative abundance shown for **(A)** Day 7, **(B)** Day 10, **(C)** Day 12. Significance between each group and its antibiotic counterpart is denoted by vertical bars. Solid horizontal bars denote significance between non-antibiotic treated groups (*, 0.01 < *P* < 0.05; **, 0.001 < *P* < 0.01; ***, 0.0001 < *P* < 0.001; ****, *P* < 0.0001; unpaired *t* test).

Supplementary Figure 6 **(A–C)** Linear discriminant analysis effect size (LEfSe) analysis between antibiotic treated and control groups on day 12 at the family level. Histogram of LDA scores plotted on a log 10 scale.
