# Supplementary material for: Coffee Consumption Modulates Amoxicillin-Induced Dysbiosis in the Murine Gut Microbiome
Source: Front Microbiol. 2021 Jun 30;12:637282. doi: 10.3389/fmicb.2021.637282 (PMC8278525; doi:10.3389/fmicb.2021.637282)
Supplement: Supplementary file 9 [file Data_Sheet_3.pdf]

Supplementary Data

Figure S1 – Phylogenetic diversity of experimental groups as measured by the Faith's Phylogenetic Diversity Index. Data are represented as mean ± standard deviation (SD). Significance was determined by the Benjamini, Krieger and Yekutieli test to correct for False discoveries with adjusted p-value < 0.05. (*, 0.01 < P < 0.05; **, 0.001 < P < 0.01; ***, 0.0001 < P < 0.001; ****, P < 0.0001). Significance between each group and its antibiotic counterpart is denoted by stars (*) with the color coordinating to control (ctrl, blue), decaffeinated coffee (decaf, purple), or caffeinated coffee (caf, pink). Significance between different experimental groups is denoted by squares color-coded according to the experimental groups being compared. Solid squares represent groups without antibiotics, open squares represent groups given antibiotics. Each comparison of two experimental groups has its corresponding significance denoted in stars (*) next to it. *n* = 6.

Figure S2 – Relative abundance of bacterial phyla over the course of the experiment. Dotted line denotes beginning of antibiotic treatment. **(A)** Control, **(B)** Control + amoxicillin days 5-12, **(C)** Decaf, **(D)** Decaf + amoxicillin days 5-12, **(E)** Caf, **(F)** Caf + amoxicillin days 5-12. *n* = 6.

Figure S3 – Linear discriminant analysis effect size (LEfSe) analysis between treatment groups on day 7 at the phylum level (**A, C, E, G, I**), and at the family level (**B, D, F, H, J)**. Histogram of LDA scores plotted on a log 10 scale.

Figure S4 – **(A-C)** Linear discriminant analysis effect size (LEfSe) analysis between antibiotic treated and control groups on day 10 at the family level. Histogram of LDA scores plotted on a log 10 scale.

Figure S5 – *Burkholderiaceae* relative abundance, with control and antibiotic samples interleaved by ranked abundance of each taxon. Means represented by dotted (ABX) or dashed (Control) lines. Relative abundance shown for **(A)** Day 7, **(B)** Day 10, **(C)** Day 12. Significance between each group and its antibiotic counterpart is denoted by vertical bars. Solid horizontal bars denote significance between non-antibiotic treated groups (*, 0.01 < P < 0.05; **, 0.001 < P < 0.01; ***, 0.0001 < P < 0.001; ****, P < 0.0001; unpaired t test).

Figure S6 - (A-C) Linear discriminant analysis effect size (LEfSe) analysis between antibiotic treated and control groups on day 12 at the family level. Histogram of LDA scores plotted on a log 10 scale.
